# Bridging annotated microscopy imaging data and analysis method development for scientific discovery

**DOI:** 10.1016/j.patter.2026.101542

**Published:** 2026-04-23

**Authors:** Kevin A. Yamauchi, Virginie Uhlmann

**Affiliations:** 1 Department of Biosystems Science and Engineering, ETH Zurich, Basel, CH, Switzerland; 2 Swiss Institute of Bioinformatics, Basel, CH, Switzerland; 3 Department of Molecular Life Sciences, Universität Zürich, Zürich, CH, Switzerland; 4 European Bioinformatics Institute (EMBL-EBI), European Molecular Biology Laboratory, Cambridge, UK

## Abstract

While modern imaging technologies offer unprecedented opportunities to observe life across scales, distilling an understanding of the underlying biological processes from these complex, high-dimensional data remains challenging. Computational analysis methods have been lagging behind our ability to produce data, as their development often requires expertise across multiple domains, including life and computer sciences. Annotated image datasets play a key role in fostering the development and improvement of microscopy image analysis methods, as they offer a realistic basis to build upon and invaluable ground truth to evaluate and optimize performance. Drawing inspiration from adjacent fields to microscopy imaging, we discuss in this perspective how sharing annotated datasets has driven progress in computational analysis. We emphasize the critical role that open data standards and infrastructure play in realizing the full scientific potential of annotated image datasets and close by highlighting opportunities for members across the scientific community to cultivate a dynamic ecosystem of data, infrastructure, and analysis methods to elevate research quality and accelerate innovation.

## The bigger picture

Since the early days of biological research, scientists have relied on microscopy images to understand how living systems function. Microscopy technologies have improved considerably in recent years, enabling scientists to capture images with unprecedented scale and information content. Modern life sciences researchers now face a conundrum: while they can routinely acquire vast amounts of microscopy imaging data, they struggle to extract the wealth of information they contain. Developing the computational methods needed to mine information from these complex images requires expertise from both biology and computer science, two communities that do not speak the same language.

## Teaser

Modern microscopy generates vast amounts of high-quality imaging data, yet extracting biological insights from these data remains challenging as it requires both productive collaboration between life scientists and computational researchers from fields such as computer and data science, as well as robust open data formats and software infrastructure. This perspective provides recommendations for all members of the scientific ecosystem to build a sustainable future that maximizes the scientific value of microscopy data.

## Introduction

Modern microscopy generates vast amounts of high-quality imaging data, yet extracting biological insights from these data remains challenging. As a consequence, although the potential of these data extends well beyond their originally intended purpose, they remain largely untapped. Addressing this requires the development of sophisticated computational methods, which in turn demands a productive collaboration between life scientists and computational researchers from fields such as computer and data science. Annotated datasets (i.e., collections of images paired with expert labels) provide a concrete mechanism to enable such interdisciplinary exchanges by giving computational researchers the resources they need to develop and benchmark new analysis methods. However, realizing the full potential of annotated datasets requires robust open data formats and software infrastructure that allow data to be correctly interpreted across different platforms and through time. We argue that strategic investment to support the production and sharing of annotated datasets and to develop their supporting infrastructure is essential to get the most value from the expensive microscopy experiments routinely carried out in modern life sciences research and to maximize their contribution to biological discovery. In this perspective, we provide specific recommendations for all members of the scientific ecosystem to build a sustainable future that maximizes the scientific value of microscopy data.

Annotated datasets (i.e., collections of images paired with expert labels identifying the information they contain) serve as a bridge between these disciplines. They allow computer scientists to develop and test novel analysis methods and, in turn, grant biologists access to new cutting-edge computational tools. For annotated datasets to fulfill this bridging role, however, they must be shared widely across research communities, shifting the challenge from creating datasets to building the infrastructure that makes microscopy image data sharing practical and sustainable. To facilitate the development of new computational analysis methods, annotated datasets must be stored in standardized formats and accompanied by supporting tools to manipulate them that are actively maintained by the community. This supporting infrastructure is essential, as it allows researchers to reap the full benefits of the considerable resources invested in generating high-quality microscopy data and speeding up the pace of biological discovery. Addressing this challenge requires coordinated action from all members of the scientific ecosystem to foster the development of a sustainable ecosystem in which the potential of imaging data for scientific discovery is fully leveraged.

Modern microscopy generates vast amounts of high-quality imaging data, yet extracting biological insights from these data remains challenging as it requires both productive collaboration between life scientists and computational researchers from fields such as computer and data science and robust open data formats and software infrastructure. This perspective provides recommendations for all members of the scientific ecosystem to build a sustainable future that maximizes the scientific value of microscopy data.

## Why annotated datasets?

Microscopy technology has developed at an extraordinary pace over the past two decades, enabling the imaging of living systems with unprecedented detail across multiple spatial and temporal scales.^1^^,^^2^ This technological revolution has transformed the life sciences by making it possible to investigate complex, dynamic biological processes in real time in their native 3D environment.^3^^,^^4^^,^^5^ Recognizing the immense potential of modern microscopy techniques, research institutions worldwide, both in the academic and industrial worlds, have been investing heavily in acquiring, maintaining, and developing cutting-edge imaging instruments. Microscopy image data analysis, however, has received comparatively little attention and resources and still too often comes as an afterthought.^6^ Consequently, life sciences research finds itself in a paradoxical situation: it routinely acquires enormous quantities of information-rich images yet only manages to exploit a fraction of the insights these data hold. This analytical bottleneck stems partly from the sheer complexity of modern microscopy data, which are massive, high dimensional, and computationally demanding to process.^7^ It is, however, also caused by the fact that the expertise required to analyze such data often resides outside of the life sciences: it is often found in adjacent communities such as computer and data science, whose researchers critically need access to well-organized and appropriately documented datasets in order to develop new computational analysis methods. As a result, advanced microscopy imaging technologies are falling short of their transformative potential: we continue to generate a flood of expensive data and underinvest in the analytical infrastructure needed to fully utilize them.

While the need for computational analysis becomes self-evident as individual microscopy image datasets grow beyond what humans could manually inspect, automated image analysis is equally valuable at smaller scales. Even when dealing with seemingly tractable datasets, computational approaches deliver a level of reproducibility and consistency that manual analysis cannot match.^8^^,^^9^ This is particularly critical in contexts where reliable, reproducible quantification is essential, such as in translational research and clinical applications. Furthermore, even when individual images are small, datasets comprising thousands or millions of them demand automated processing. The ability to analyze such large collections computationally, a task that would be prohibitively time-consuming for humans, increases the statistical power of observations and leads to more robust conclusions.^10^ Ensuring that microscopy images are analyzed with the latest computational methods is therefore not solely a matter of convenience but a prerequisite to maximizing the quality and reliability of scientific findings.

Given the enormous realized and opportunity costs associated with preparing, imaging, and processing high-quality microscopy data, we argue that these valuable resources should be computationally mined to be used to their full potential. One approach to preventing data waste and facilitating further analysis is to share datasets in an open and FAIR (findable, accessible, interoperable, and reusable^11^) manner. This is enabled by the creation of data archives^12^^,^^13^^,^^14^ and support frameworks.^15^^,^^16^^,^^17^ However, in practice, these efforts primarily benefit researchers within the community that generated the data. A key challenge posed by microscopy data is that unlocking their full potential as a source of quantitative information requires the development of novel, dedicated computational approaches and, therefore, the combined expertise of multiple disciplines.^18^ Indeed, while computer vision has advanced dramatically over the past two decades through the advent of artificial intelligence (AI), the algorithms developed in this field are designed for everyday photographs and videos, which significantly differ from microscopy data. Many of the assumptions underlying these methods (e.g., object sizes affected by perspective and fixed orientation due to gravity) do not hold in microscopy, while challenges specific to biological imaging (e.g., densely packed and morphologically similar objects, low signal-to-noise ratios, and cell division events) are not accounted for. Therefore, these methods often cannot be directly applied to biological images: they can serve as a foundation for developing microscopy-specific approaches, but this requires dedicated research and specialized computer vision expertise that life scientists typically do not possess. A productive collaboration between fields is mutually beneficial: life scientists are in dire need of sophisticated strategies to extract meaning from big image data, while computer vision researchers are keen to tackle the exciting and novel computational challenges unique to microscopy and biology.

Annotated datasets provide a simple yet powerful mechanism to catalyze these interdisciplinary interactions. They pair primary data (e.g., images) with additional information that provides context for their interpretation. Annotations that are useful for biological research may take many forms (Table 1), including spatial annotations that assign meaning to specific image regions (e.g., segmentation masks delineating cell nuclei), sample annotations that describe the biological content of the specimen (e.g., disease state or phenotype classifications), and instrument metadata that record the acquisition parameters (e.g., optics and illumination settings). These annotations are essential for interpreting the data and assessing whether and how they can be reused in subsequent studies. For computational method developers, they are particularly critical, as they often serve as the ground truth against which algorithms are trained and evaluated. The availability of curated, high-quality annotations is what transforms a dataset from an opaque “data dump” into a resource of tremendous scientific value. A compelling example from outside of microscopy imaging can be found in the structural biology community, where curated resources such as the Protein Data Bank,^19^ enriched with sequence and functional annotations from databases such as UniProt,^20^ laid the foundation for the revolutionary AlphaFold algorithm.^21^ Within microscopy image analysis, recent breakthroughs in the extremely challenging problem of 3D cell tracking^22^^,^^23^ were made possible by the availability of a rich corpus of annotated data spanning diverse contexts (e.g., 2D and 3D, different imaging modalities, and different organisms). Building on annotated datasets assembled by the life sciences community, these advances enabled purely computational researchers to develop novel algorithms that address a long-standing challenge. This is especially true for AI-based methods, where annotated datasets are not merely helpful but indispensable, as exemplified by the automation breakthroughs achieved in computer vision by AI algorithms trained on a large corpus of annotated data.^24^ Therein lies a critical automation gap in microscopy: the ability of supervised AI methods to generalize accurately is a direct function of the quantity and quality of annotated datasets available for training. Closing this gap through efforts to produce and share annotated datasets would therefore turbocharge method development and substantially contribute to the advent of novel analytical tools needed to fully harness the rich information held in modern microscopy images. Moreover, as multiple competing analysis methods emerge and evolve, annotated datasets enable their objective comparison and make it possible to assess relative performance and identify respective strengths and limitations. This benchmarking process is central to method validation and improvement, as illustrated by the instrumental role that datasets such as ImageNet,^25^ MS-COCO,^26^ and CIFAR,^27^ among many others, have played in advancing analysis techniques for the images classically considered in computer vision (e.g., photographs of human faces, street scenes, animals, and everyday objects) and driving decades of progress in the field.

**Table 1:** Common types of annotations in microscopy image datasets

Annotation type	Description	Examples
Spatial	labels assigning meaning to specific image regions	segmentation masks (e.g., cell nuclei and membranes), bounding boxes around objects of interest, point annotations of cell centroids, anatomical landmarks
Temporal	labels linking objects or events across time points (often referred to as “tracks”)	cell lineage trees, identifiers linking cells across frames, event labels (e.g., division and apoptosis)
Sample metadata	labels providing information on the biology of the specimen present in the data	cell type, tissue identity, disease state, phenotype classification, treatment condition
Instrument metadata	parameters providing information on how the data were acquired	microscope type, objective magnification and numerical aperture, illumination wavelength and intensity, detector settings, physical pixel size
Quality	labels providing information on the data quality or reliability	confidence scores, imaging artifacts flags, quality control metrics

While highly valuable, creating annotated datasets is both challenging and laborious. To be truly useful, annotations must be complete and high-quality, which demands considerable time and expertise. Given the wide range of annotation types one could include, knowing what to prioritize for a given dataset is itself a non-trivial question. The perceived (and real) cost of determining what to annotate, how to do it, and actually carrying it out is often so substantial that it becomes overwhelming, particularly for resource-limited laboratories. To make this more tractable, communities have begun defining standards for specific use cases. Several consortia^28^^,^^29^^,^^30^ have developed guidelines for the various annotations that should accompany imaging data, providing tiered frameworks that distinguish essential information from desirable or gold-standard metadata and, equally importantly, helping identify which annotations may not be relevant. In addition, two recurring essential ingredients of a useful annotated dataset are a unique identifier that allows it to be directly referenced and a license with clear rules for usage. While these guidelines clarify what should be recorded, collecting and storing metadata in a compliant manner remains a practical challenge for life scientists. Recent efforts aim to alleviate this burden through software tools that automate the collection and formatting of instrument metadata^31^ or through intuitive interfaces that enable collective manual annotation, curation, and editing.^32^ Complementary initiatives from scientific publishers and public archiving repositories seek to address the incentive problem directly by providing formal recognition for the effort invested in producing and sharing high-quality annotated datasets. Finally, since defining which annotations are needed will always resist a universal answer, it is practically most useful to acknowledge that creating generic annotated datasets that serve all conceivable downstream tasks is unrealistic. Annotated datasets are most valuable when designed with a specific analytical purpose in mind, ensuring that critical annotations for that purpose are included.

## Annotated datasets are an entry point for multidisciplinary collaboration

**Figure 1:**
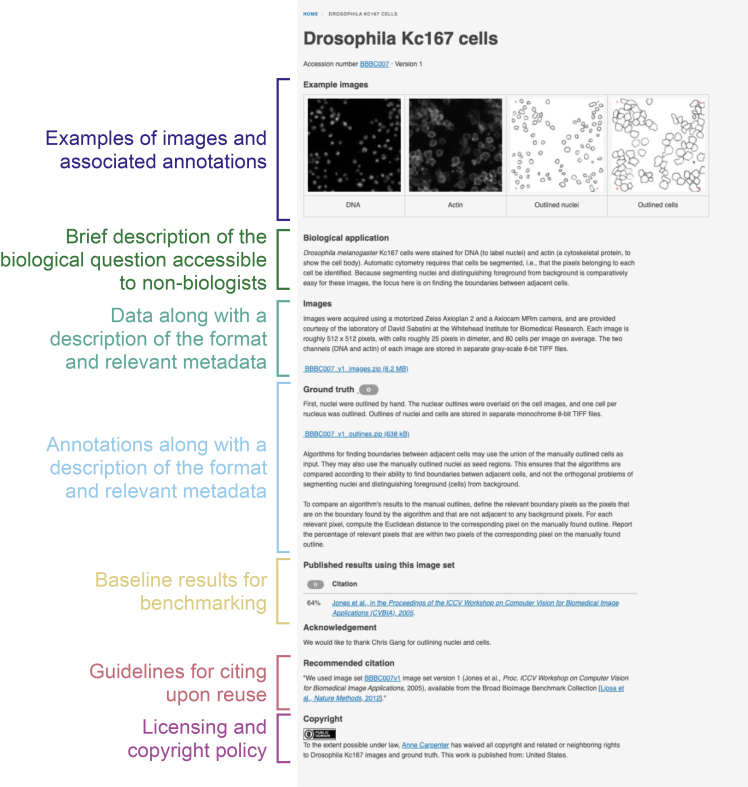
Anatomy of a reusable annotated dataset Screenshot from the Broad Bioimage Benchmark Collection dataset entry BBBC007 (https://bbbc.broadinstitute.org/BBBC007) highlighting features that facilitate the reuse and interpretation of annotated datasets. This example illustrates how contextual information can help go beyond mere availability and enhance reuse by enabling researchers to quickly assess the relevance of a dataset, understand the structure and content of the data, and integrate it into the development of new analysis methods.

The potential of annotated datasets to lower barriers for computational scientists to make meaningful contributions to biological discovery is further demonstrated by the comprehensive ecosystem of well-defined computational problems, standardized evaluation metrics, and curated annotated datasets assembled by the Open Problems in Single Cell Analysis initiative.^33^ While microscopy imaging lacks such a unified platform, several exemplary individual initiatives demonstrate the tremendous potential of well-curated annotated datasets. The Broad Bioimage Benchmark Collection (BBBC^34^), established in 2012 to address the lack of standardized evaluation resources tailored to bioimage analysis methods, has pioneered the systematic comparison of object detection, segmentation, and classification algorithms across biological contexts and imaging modalities (Figure 1). One of the revolutionary aspects of the BBBC that still makes it stand out today is the inclusion of simplified explanations of the biological applications underlying each dataset, alongside the image data and ground-truth labels provided in easy-to-use formats. This biological context is crucial for allowing non-biologists to understand the bigger picture beyond merely recovering labels, thus transforming abstract computational tasks into meaningful scientific problems. This thoughtful design has contributed massively to the popularity and impact of this dataset collection, demonstrating how accessible resources can bridge disciplinary divides. Similarly, the 2018 Kaggle Data Science Bowl brought global attention to nucleus segmentation, prompting thousands of participants to develop innovative approaches to this fundamental bioimage analysis challenge.^35^ Nucleus segmentation is the process of delineating the contours of individual cell nuclei and constitutes a ubiquitous step in microscopy image analysis workflows, as it is a prerequisite to quantifying nuclear size, morphology, or spatial organization within a tissue or cell population. The competition not only advanced the state of the art in nucleus segmentation but also demonstrated the power of community-driven method development when provided with high-quality annotated data. Industrial stakeholders are also key contributors to annotated microscopy datasets. The recent LIVECell dataset,^36^ produced by the microscopy instrument company Sartorius, offers the largest-to-date collection of label-free light microscopy images of cells from a diverse set of cell morphologies and culture densities, with over 1.6 million objects along with their corresponding segmentation masks provided in the standardized MS-COCO object detection format. This resource has become a gold standard for training deep-learning-based cell segmentation methods and has already contributed to the design of generalist^37^ and foundation^38^ models with high reusability potential. It illustrates how industry can make substantial contributions to the annotated dataset ecosystem, driven by a shared interest in advancing the computational methods that underpin their own products and services.

Beyond static images, the Cell Tracking Challenge has been continuously pushing the capabilities of analysis methods for cell tracking in time-lapse microscopy data since 2013.^39^ This initiative, providing data across diverse samples and imaging modalities encompassing both 2D and 3D datasets as well as synthetic and real microscopy images, has driven the definition of a standardized and open format for annotating tracks as well as the elaboration of novel biologically informed performance evaluation metrics tailored to the specificity of cell-tracking problems. These tailored tracking metrics complement the classical computer vision ones and provide a more comprehensive assessment of performance in microscopy data, allowing comparison of the relative performance of novel computational methods as they come out.^40^ In addition to successfully supporting and incentivizing the development of better computational methods to solve one of bioimaging’s key problems, the Cell Tracking Challenge therefore demonstrates how annotated datasets can drive the definition of standardized performance measurements that are both computationally rigorous and biologically meaningful.

One additional challenge is that different researchers and communities may require different annotations for the same dataset. This is especially pertinent for label-free imaging techniques such as volume electron microscopy (vEM) and cryo-electron tomography (cryo-ET), where a single image captures information about the physicochemical properties of the sample rather than specific structures as in targeted fluorescence imaging methods (e.g., immunofluorescence or fluorescent fusion proteins). While this makes such image data extraordinarily information rich, it also renders the annotation task daunting. Producing a fully segmented dense volume is so labor-intensive as to be humanly impossible.^41^ Crowdsourcing and iterative annotation are therefore essential but introduce their own challenges around provenance, versioning, and preventing fragmentation as multiple contributors add and refine annotations over time. Members of the cryo-ET community have addressed this by creating the CryoET Data Portal,^42^ a public resource that enables new annotations to be contributed to existing datasets. The portal provides clear guidelines for uploading annotations of existing data,^43^ thereby encouraging collaborative community efforts. A large ground-truth dataset for particle detection in cryo-ET images was assembled using these standardized image and annotation formats. Together with open-source software for loading these data into common deep learning frameworks,^44^ these annotated data served as the basis for a Kaggle challenge on improving particle detection.^45^^,^^46^ This competition engaged over 1,000 participants and resulted in new state-of-the-art models,^46^ highlighting the value of standardized data formats with open software infrastructure for developing new methods. The recent *Chlamydomonas reinhardtii* dataset^47^ further exemplifies collaborative annotation in cryo-ET, bringing together researchers from academia and industry to jointly tackle the challenge of annotating a complex cellular volume, which, in turn, enabled new biological discoveries^48^ and has already been used for developing and testing new segmentation and analysis methods.^49^^,^^50^

Although these examples offer clear examples of the value of annotated datasets, they cover only a fraction of the challenges encountered in microscopy image analysis. Making sense of microscopy images indeed entails far more than identifying objects and following them over time. As optical microscopy images are inherently diffraction limited and noisy, image restoration constitutes an important first step of microscopy image analysis,^51^^,^^52^ along with the fundamental questions it raises about distinguishing genuine biological features from computational artifacts in restored images.^53^ Similarly, the complexity and multiscale nature of biological architecture make virtual labeling (i.e., the process of computationally predicting how a sample labeled with a specific marker would appear, without physically performing the labeling and imaging) and computational super-resolution (i.e., the process of inferring a super-resolved image from a diffraction-limited one) particularly relevant to microscopy image analysis.^54^^,^^55^ The prospect of robust algorithms for these tasks offers valuable opportunities to optimize experimental costs and timelines. By identifying cases that are the hardest to predict computationally (e.g., those yielding the lowest confidence or highest uncertainty), these methods can help researchers target costly experimental interventions, such as additional labeling or super-resolution imaging, where they are most needed rather than applying them indiscriminately. While promising, these methods introduce the challenge of validating their outputs and assessing the extent to which they can be trusted. Finally, it is worth noting that segmented objects are never the end goal of microscopy image analysis, which ultimately seeks to understand the underlying biological processes and mechanisms. How to extract meaningful quantitative representations of objects, often over time, to achieve this understanding and how to evaluate the relative merit of different representations remain entirely open questions for which reference problems have yet to be proposed. All of these open challenges would benefit tremendously from annotated datasets and benchmarking standards, not only to drive innovation but also to help the life sciences community navigate the recent proliferation of algorithms proposed to address them. The creation and sharing of annotated datasets is therefore a necessary strategic investment. It is, however, not sufficient to spark methodological innovations and yield returns for the broader scientific community: to be useful, annotated datasets must be supported by standardized data formats and software infrastructure facilitating their manipulation.

## Open formats and infrastructure enable discovery from data

**Figure 2:**
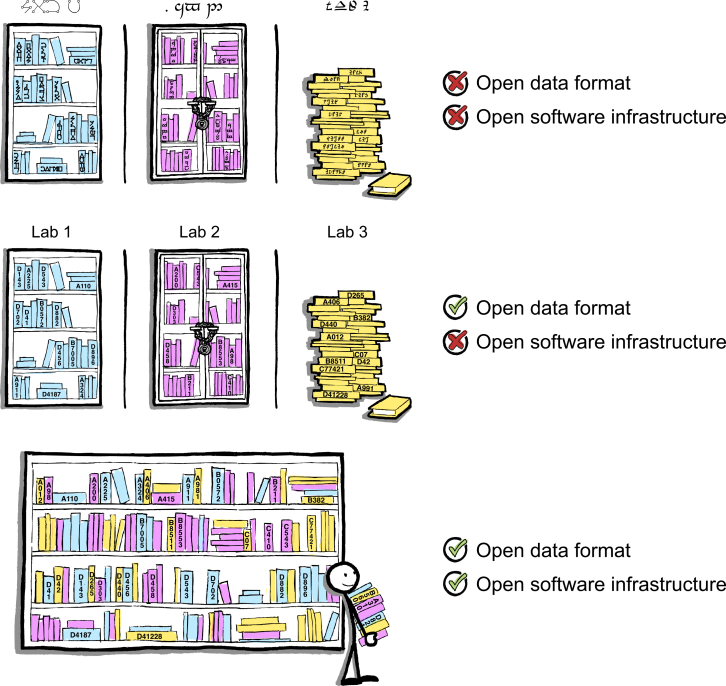
Open data formats and software infrastructure support the exploration and integration of biological data The challenge of exploring and integrating information contained in microscopy data of diverse provenance can be illustrated by analogy with drawing information from various books. Without open data formats or open software infrastructure (top), each lab stores its data in formats, akin to books written in different languages and kept on isolated, sometimes inaccessible shelves. Data cannot be easily read, compared, or combined. When open data formats are adopted but open software infrastructure is lacking (middle), datasets become mutually intelligible, akin to books written in a common language, and can be labeled and cataloged consistently. However, without shared infrastructure, the data remain scattered across separate collections, some behind locked doors, making systematic discovery and integration difficult. When both open data formats and open software infrastructure are available (bottom), all datasets can be brought together into common, openly accessible libraries. Researchers can then freely browse, retrieve, and combine resources from different provenances, enabling efficient exploration and integration that supports novel insights.

Although making annotated microscopy image datasets openly available is a challenge in its own right, it is not sufficient to realize their potential for reuse. For the data to be useful, researchers must be able to correctly load them into computational pipelines and to make sense of their content. The key ingredients connecting microscopy imaging data to biological insights are standardized data formats and robust software infrastructure for reading and writing these data (Figure 2).

Data formats provide a unified approach to storing both the primary data (such as images, sinograms, etc.) and the critical metadata and annotations required for their interpretation.^56^^,^^57^^,^^58^ The choice of format impacts not only the types of data that can be stored but also the performance of access operations, the latter being an increasingly crucial consideration as datasets grow ever larger. Software infrastructure provides implementations for reading and writing data in the form of standardized methods for accessing and interacting with microscopy images. As a result, software infrastructure enables the seamless integration of datasets from different sources and experimental conditions.^14^^,^^59^ The more standardized, well-documented, and stable these formats and infrastructure components are, the lower the barriers for researchers from adjacent computational fields to contribute their expertise to biological discovery. The importance of unified standards is particularly acute in microscopy image analysis. Microscopy technology evolves continuously, driven by new biological questions: new instruments appear regularly, each producing data in its own format optimized for the specific hardware’s writing speed and efficiency. In the absence of a unified infrastructure for reading these formats, computational analysis methods must be painstakingly adapted for every new data type that emerges. A striking illustration of this challenge is the open-source Bio-Formats library,^60^ which, since its creation in 2006, has continually expanded its support for microscopy file formats, encompassing over 140 at the time of writing.^61^ Bio-Formats has become one of the standard ways bioimage analysts are able to read and write image data, as it provides file I/O (input/output) and conversion in many common bioimage analysis and visualization tools, such as ImageJ,^62^ Fiji,^63^ CellProfiler,^64^ Imaris, Micro-Manager,^65^ Icy,^66^ Qupath,^67^ and MATLAB.^68^ However, without a common open standard onto which these diverse formats can be mapped, maintaining such an infrastructure to ensure that it keeps pace with the constant influx of new formats is virtually unsustainable. As a result, some formats (and therefore datasets) are inevitably left inaccessible.^69^ To truly harness microscopy imaging data holistically and analyze them with the latest available tools, both open data formats and open software infrastructure are therefore indispensable.

Community-driven efforts have emerged across many scientific domains to standardize data formats and software infrastructure for FAIR data access. The single-cell analysis community is a positive example of a community building analysis tools and data resources around open standards to drive biological discovery via productive collaboration between computational and experimental life scientists.^70^^,^^71^^,^^72^ The scverse community^71^ has developed data structures and formats, such as AnnData^58^ and SpatialData,^73^ that serve as points of interoperability for analysis libraries. By standardizing around the data infrastructure, analysts can compose workflows from analysis libraries across this ecosystem. It also makes access to large data resources, such as the CELLxGENE portal,^74^ and benchmark datasets, such as the Open Problems in Single-Cell Analysis,^33^ seamless. Complementary efforts, such as SpatialFeatureExperiment,^75^ have been carried out in the R community using the Bioconductor project.^72^ Importantly, there is an ongoing push to bridge data standards across existing programming language divides.^76^

As data formats and software infrastructure specify what data can be stored and how they are accessed, they must remain open and community driven. Openness is crucial to ensure transparency and to allow everyone to contribute. Proprietary formats and software, on the contrary, hamper interoperability and exacerbate inequitable access. It is also critical that the governance of open data formats and software infrastructure remains with the community, as these essential resources are living bodies of work that must be free to continuously evolve to meet the needs of cutting-edge research, even when they do not necessarily align with short-term commercial interests. The emergence and sustained success of community-driven initiatives, such as AnnData and Bioconductor, demonstrate a clear consensus within the scientific community about the value of open, standardized infrastructure: through their adoption patterns, researchers have revealed a strong preference for these community standards over proprietary commercial solutions. This grassroots adoption reflects not only preference but also necessity: only open, community-governed tools can provide the stability, interoperability, and long-term accessibility that rigorous science demands. Further evidence of the single-cell analysis community choosing community-driven analysis methods and standards over commercial solutions is provided by companies such as 10× Genomics recognizing Bioconductor and scverse tools as the “most popular ecosystem” for analyzing spatial omics data.^77^ The fact that these initiatives have flourished in spite of headwinds from institutional and funding pressures underscores both the depth of need and the impact of open infrastructure. Interestingly, open formats and infrastructure also benefit industrial stakeholders, who increasingly depend on community-developed resources for their own innovation. Several microscopy instrument manufacturers already integrated community-developed, open-source analysis methods such as cellpose^37^ into their commercial platforms, while pharmaceutical companies increasingly rely on openly shared annotated datasets to train models for drug discovery. These examples underscore a virtuous cycle: open infrastructure enables industry innovation, which in turn generates resources and demand that strengthen the ecosystem as a whole.

Since data formats and software infrastructure must evolve to keep up with the needs of the scientific community, their design requires continual maintenance and updating. As long as microscopy instrument manufacturers and software developers continue creating proprietary formats, substantial efforts will be needed to maintain unified standards that bridge these disparate systems.^60^ This presents a significant challenge, as few resources are typically allocated to support these essential yet research-adjacent infrastructure efforts.^78^^,^^79^ While annotated datasets are a crucial first step toward developing better and more powerful microscopy image analysis methods, investing in the infrastructure supporting their use is the next critical frontier. Just as with curating and sharing annotated datasets, appropriately resourcing infrastructure development represents a strategic investment that magnifies the impact of both microscopy data generation and analysis method development efforts and ensures that expensive microscopy imaging experiments translate into meaningful biological discoveries.

## Moving forward together toward a more holistic approach to life sciences

**Figure 3:**
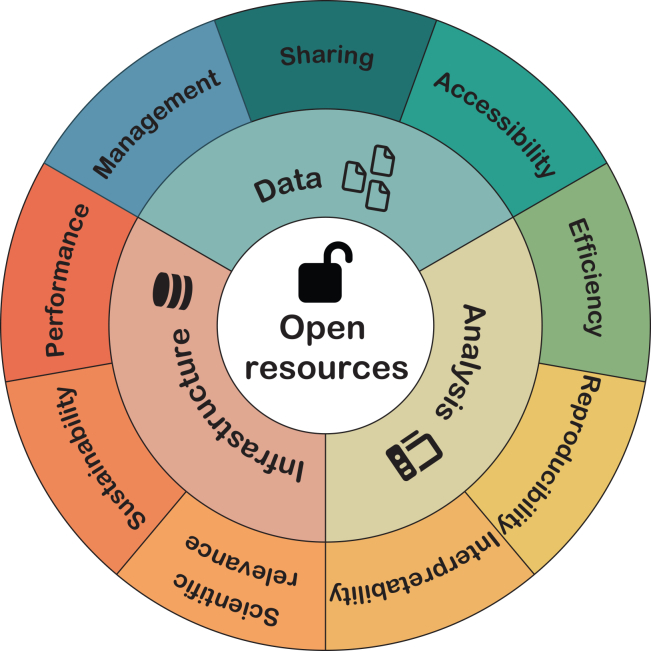
Open resources elevate all aspects of scientific research On the data front, open resources facilitate data management and sharing and improve their accessibility across different research communities. On the analysis side, open resources reduce the risk of redundant tool development, enable reproducibility, and promote interoperability, thereby contributing to more accurate and robust analysis software. Finally, open infrastructure supports the pursuit of relevant science through community input, ensures the long-term sustainability of essential scientific tools, and improves the quality of research through collaborative optimization. While our discussion focuses on image data analysis, open resources can equally benefit image data acquisition, for instance, through open hardware initiatives, ultimately leading to more reliable and precise instruments and therefore higher-quality images.

Excellent research is fundamentally dependent on high-quality reference datasets, which can only realize their potential when supported by robust open formats and infrastructure. In an ideal world, we envision a seamless ecosystem that enables microscopy image data to flow freely across its entire life cycle from acquisition to analysis and ultimately sharing and across the full spectrum of research domains, institutions, and scientific questions (Figure 3). Such a truly integrative culture of science would enable experiments, analysis, interpretation, and hypothesis formulation to be tightly coupled through an open, community-driven infrastructure that would be stable, widely adopted, and continuously evolving to meet the needs of research.

Significant barriers, unfortunately, still stand in the way of realizing this vision. First, the siloed structure of academic research, constrained by departmental boundaries and project-focused incentives, hinders opportunities to maximize the value and impact of scientific outputs beyond the scope of individual projects.^80^ It is important to emphasize that the technical implications of sharing data, annotated or raw, are not the barrier *per se*: scientists have been sharing data informally through exchanges of physical hard drives, direct transfers, and institutional repositories for decades. The real challenge lies in making this sharing robust, sustainable, and genuinely useful by ensuring that data can be correctly interpreted years later by researchers using different approaches and investigating different questions. This endeavor requires standardized, open formats that preserve both the data and metadata, as well as software infrastructure that remains maintained. Unfortunately, open software infrastructure remains chronically underfunded despite the significant economic value it generates,^81^ resulting in exceptionally skilled developers stretched thin and often left in precarious positions due to the lack of funding streams that support long-term sustainability. This combination of poor working conditions and career prospects too often results in loss of expertise and talent that are essential to support and enable novel academic research. Perhaps most concerning, many scientists may not fully realize their dependence on the open infrastructure enabling their work. As a result, research institutions dramatically miss out on the benefits of a synergy between open infrastructure development and scientific novelty. The consequences of the disconnect between the true and acknowledged importance of open infrastructure are perhaps best evidenced by the previously mentioned BioFormats^60^ tool for reading proprietary image formats. This open infrastructure is routinely used by life scientists worldwide in their daily research practice, often without them consciously realizing it, and its sudden disappearance would have a catastrophic impact on scientific progress.

While greater investment in open formats and infrastructure would already go a long way, it would not suffice to truly bridge the divide between data generation and computational analysis to realize the full potential of microscopy for life sciences discovery. Connecting different scientific communities requires more than shared technical resources: it demands a common language, which takes sustained effort and time to build.^18^ In the absence of such a language, life scientists will continue to lack clarity on which annotations should accompany a dataset to make it genuinely useful for method developers, while computational researchers will continue to have a limited understanding of what may constitute a practical and valuable tool from a biologist’s perspective. Although no simple roadmap exists for achieving this, examples from other fields offer valuable inspiration. In neuroimaging, the Brain Imaging Data Structure emerged from a collaborative effort between experimentalists and computational researchers to define a shared standard for organizing and annotating brain imaging data and has since become a cornerstone of reproducible research in the field.^82^ Similarly, the Human Cell Atlas consortium brings together biologists, clinicians, and computational scientists under a governance framework that aims to facilitate the development of shared standards and resources.^83^ These examples illustrate how appropriate governance models bringing representatives from different communities together around concrete, shared objectives can help forge the common language needed to set mutual expectations and find productive common ground.

The emergence of next-generation file formats for microscopy data^57^^,^^84^ offers a rare opportunity for international coordination in the microscopy imaging space. Unlike classical image formats, which store data in a monolithic, sequential manner, next-generation file formats decompose images into a multitude of small chunks that are logically organized to represent the whole dataset. This chunked architecture enables selective access: only the portion of an image relevant to a given task needs to be loaded and processed at any one time. Such capabilities are critical when working with data in the terabyte range or beyond, as commonly generated by modern microscopy systems. They make it possible, for instance, to process images that far exceed a computer’s active memory or to stream individual data chunks from a remote file rather than transferring it in its entirety. As this new open standard develops and becomes established, researchers who fail to adopt it will increasingly lock themselves out of the latest technological innovations in microscopy imaging and image analysis, ultimately compromising their competitiveness. Likewise, annotated microscopy datasets have an important role to play in realizing the enormous potential of a community that aligns around shared infrastructure and standards. The entire research ecosystem can contribute to this effort, with each stakeholder group playing its part in laying the foundation that will enable the next generation of biological discoveries. In that spirit, we outline the following recommendations.

• When sharing new microscopy image datasets, whether annotated or not, experimental researchers should prioritize building upon existing open formats and infrastructure rather than developing isolated solutions.
• Computational researchers should strive to actively reuse annotated datasets to validate and benchmark analysis methods. When existing open formats or infrastructure fail to meet their needs, they should engage constructively with the communities developing them rather than creating parallel solutions to allow open tools to evolve and address real-world needs.
• When their research benefits from open infrastructure, which will almost always be the case, principal investigators should take actions to support its continued development and maintenance. This can be achieved in many different ways, from direct financial contributions to the maintainers of the infrastructure to allocating personnel time to contribute to these efforts.
• Primary infrastructure maintainers should structure their governance framework and codebase to welcome contributions from diverse backgrounds and create clear pathways for community involvement. They should also foster responsive and inclusive development practices.
• Academic institutions should develop mechanisms that facilitate and reward contributions to open infrastructure, recognizing that such investments are beneficial to their scientific enterprise as a whole. Opportunities to achieve this include supporting researchers and facility staff in contributing to open resources and hosting community events such as hackathons,^76^^,^^85^ among many others.
• Microscopy instrument vendors should strive to support open formats and infrastructure by providing tools to either write data and associated metadata directly in standardized open formats or to convert proprietary formats into them. Vendors should also engage with the communities developing open infrastructure to ensure that their own data formats remain compatible with evolving community standards, thereby significantly lowering the burden on researchers for sharing, reusing, and analyzing data across platforms.
• Funding agencies should recognize infrastructure maintenance and development as a distinct form of scientific contribution by establishing dedicated funding streams that support sustainable maintenance alongside novel research. These programs should have durations and renewal criteria that align with the reality of the long-term availability requirements of scientific resources, acknowledging that maintaining robust infrastructure is itself an investment that enables discoveries.
• Finally, scientific publishers should move beyond only requiring data to be made openly available. Instead, they should aim to enforce that data are shared in open formats and that computational methods are compatible with open infrastructures, thereby incentivizing the adoption of community standards.

Importantly, we must remember that annotated datasets, as essential as they are to enable novel discovery, do not directly yield biological insights. They are instead enablers that turbocharge the development of the tools required to generate those insights. Increasing the pace of scientific progress requires moving beyond collecting data and developing methods in isolation toward intentionally combining data and analysis methods to drive new biological discoveries.

The complex challenges facing modern biological research cannot be solved through isolated efforts. They necessitate authentic multidisciplinary collaboration and close exchanges between those acquiring digital data and those developing computational analysis methods. Shared annotated microscopy datasets and robust open infrastructure facilitate these connections. In an era of economic constraints and uncertain continuity of resources, we collectively cannot afford the waste that comes from duplicated research efforts and incompatible approaches. Assembling and sharing annotated datasets, like all community-benefiting initiatives, is an incredibly efficient way of multiplying the impact of resources initially allocated to individual research groups. By committing to make their outputs accessible to the broader scientific community, researchers can help ensure that scientific progress continues unimpeded by global economic pressures^86^ while safeguarding a sustained pace of discoveries that benefit human society as a whole. The path forward requires recognizing that the most transformative discoveries emerge not from individual brilliance working in isolation but from communities working together openly with shared tools, standards, and a vision toward common goals.

## Acknowledgments

V.U. acknowledges funding from the 10.13039/501100006447University of Zurich. K.A.Y. acknowledges funding from 10.13039/501100003006ETH Zurich and the Chan Zuckerberg Initiative DAF, an advised fund of the 10.13039/100000923Silicon Valley Community Foundation (grant no. 2023-323350). The authors are grateful to excellent public infrastructure, such as the Schweizerische Bundesbahnen, for making joint writing sessions possible while preparing this manuscript.

## Author contributions

K.A.Y. and V.U. conceived the article, drafted the paper, and edited it.

## Declaration of interests

The authors declare no competing interests.
